# Integrative roles of transforming growth factor-α in the cytoprotection mechanisms of gastric mucosal injury

**DOI:** 10.1186/1471-230X-6-22

**Published:** 2006-08-01

**Authors:** Takashi Kosone, Hitoshi Takagi, Satoru Kakizaki, Naondo Sohara, Norio Horiguchi, Ken Sato, Masashi Yoneda, Toshiyuki Takeuchi, Masatomo Mori

**Affiliations:** 1 Department of Medicine and Molecular Science, Gunma University Graduate School of Medicine, Maebashi 371-8511, Japan; 2 Department of Molecular Medicine, the Institute for Molecular and Cellular Regulation, Gunma University, Maebashi 371-8512, Japan; 3 Department of Gastroenterology, Dokkyo University School of Medicine, Tochigi 321-0293, Japan

## Abstract

**Background:**

Transforming growth factor α (TGFα) protects against gastric mucosal injury and facilitates wound healing. However, its overexpression is known to induce hypertrophic gastropathy resembling Menetrier's disease in transgenic (TG) mice on an FVB background, as one of the authors reported previously. We studied another TGFα-expressing mouse line on a CD1 background, whose gastric mucosa appears normal. Since this TG mouse had a strong resistance to ethanol-induced gastric injury, we considered the long-term effect of TGFα on several gastric protection mechanisms.

**Methods:**

TGFα-expressing transgenic (TG) mouse lines bearing human TGFα cDNA under the control of the mouse metallothionein gene I promoter were generated on a CD1 mouse background, and analyzed their ethanol injury-resistant phenotypes produced by TGFα.

**Results:**

In the TG mucosa, blood flow was well maintained after ethanol injury. Further, neural and inducible types of NO synthases were consistently and widely expressed in the TG mucosa, compared with the limited distribution of neural type NO synthase in the luminal pit region of the wild-type (WT) mucosa. COX-2 and its upstream transcription factor NfkB were constitutively elevated in the TG mucosa even before ethanol administration, whereas they were induced in the same region of the WT mucosa only after ethanol injury. Two anti-apoptotic proteins, HSP70 and Bcl-2, were upregulated in the TG mucosa even before ethanol administration, while they were not expressed in the WT mucosa before the injury. Furthermore, pro-caspase 3 activation was inhibited in the TG mucosa, while it was converted to the active form in the WT mucosa following ethanol administration.

**Conclusion:**

We conclude that TGFα maintains the gastric mucosal defense against gastric injury by integrating other cytoprotective mechanisms.

## Background

Ethanol has long been used to generate a hemorrhagic gastric mucosal injury in rodents [1]. With oral administration of absolute or hydrochloric acid-acidified ethanol, extensive hemorrhagic lesions can be produced in the gastric mucosa. This experimental gastric injury model has been frequently used for gastric mucosal protection and restitution studies. Indeed, a number of cytoprotective compounds have been tested to evaluate their potency in resisting gastric injury, including gastrointestinal hormones, growth factors, prostaglandins, bioactive amines, and mild irritants such as capsaicin [2-5]. Among these compounds, epidermal growth factor (EGF) family growth factors, notably transforming growth factor α (TGFα), have been reported as potent cytoprotective compounds against ethanol-, acetic acid-, or aspirin-induced gastric injury [2,4].

TGFα was expressed in the rat gastric mucosa after oral administration of acidified taurocholate or hydrochloric acid, suggesting its reparative role in gastric injury [6]. TGFα given intraperitoneally protected against ethanol-induced gastric injury with a significant increase in adherent gastric mucin in rats [7]. The cytoprotective function of TGFα appeared to be mediated by activation of phospholipase C-γ1, but not by prostaglandins, and was independent of the anti-secretory effect of TGFα [7]. On the other hand, prostaglandins and their synthetic enzyme, cyclooxygenase (COX), have long been proposed as cytoprotective factors against gastric injury [5,8]. EGF has been shown to express an inducible COX isoform, COX-2, in the RGM1 rat gastric mucosal cell line through an ERK MAP kinase pathway [9] and in Swiss 3T3 fibloblasts, especially by co-stimulation with gastrin [10]. Furthermore, COX-2 expression by heparin-binding EGF-like growth factor, a member of the EGF family growth factors, was blocked by a specific EGF receptor (EGF-R) inhibitor in the rat gastric mucosa [11]. Thus, the EGF-R signal appears to mediate COX-2 induction in the gastric mucosa.

The most important cytoprotective mechanisms include gastric mucosal blood flow. EGF and TGFα have been shown to exert cytoprotective effects via stimulation of capsaicin-sensitive neurons with release of calcitonin gene-related peptide (CGRP) and nitric oxide (NO) in rats exposed to orogastric ethanol administration, resulting in an increase in gastric mucosal blood flow [11,12]. In contrast, Konturek et al. minimized the role of the CGRP-dependent increase in gastric blood flow by demonstrating that the administration of NO-releasing aspirin offered protection against absolute ethanol-induced gastric injury even in capsaicin-denervated rats, resulting in the upregulation of heat shock protein 70 (HSP70) expression and attenuation of the oxidative injury by strong upregulation of antioxidative genes such as zinc copper superoxide dismutase (SOD) and glutathione peroxidase [13].

TGFα was immunostained predominantly in GSM cells along the gastric pit in humans [14], intensively in gastric surface mucosal (GSM) cells over the proliferative zone and weakly along the glandular region under the proliferative zone in rats [15], and markedly along the mucous neck cell region in FVB/N strain mice [16]. On the other hand, EGF-R was immunostained in GSM cells lining the foveolar pit and mucous neck cell region to the lower glandular region in humans [17]. In rats, EGF-R was localized at the top-pit region and in the chief cell clusters at the bottom of the gastric glands [15]. Although TGFα is known to induce cell proliferation in primary-cultured rat gastric mucosal cells [18], and hyperplastic proliferation in the mucosa on the TGFα-expressing transgenic (TG) mouse [19,20], the distribution of TGFα and EGF-R in terminally differentiated gastric mucosal cells suggests that TGFα exerts non-proliferative functions including anti-apoptotic effects and gastric epithelial barrier formation in an autocrine/paracrine manner. For example, TGFα inhibited apoptosis of a mouse gastric mucosal cell line, GSM06, by expressing anti-apoptotic Bcl-2 family proteins through an NF-κB-dependent pathway [21]. Gastric apical and basolateral EGF-R has been shown to induce a decrease in paracellular permeability to acid for the protection of glandular cells in an acidic environment [18,22]. Thus, TGFα exerts both proliferative and non-proliferative effects on gastric mucosal functions, and these diverse TGFα effects should be re-evaluated, at least in terms of whether their results are derived from short-term treatment using culture cell lines or from long-term expression using TGFα-transgene-expressing animals.

A decade ago, one of the authors, H. Takagi, generated TGFα-expressing TG mouse lines under the control of the metallothionein gene promoter, and one of these lines (MT100) exhibited hypertrophic gastropathy resembling Menetrier's disease, as similarly shown by other investigators [19,20]. In contrast, another TG line (MT42) displayed a normal-appearing gastric mucosa, although TGFα transgene was expressed to a similar extent in both TG lines [20]. Since the MT42 line displayed a surprising level of resistance to ethanol-induced injury to the gastric mucosa, we considered that it could be useful for exploring the non-proliferative effects of TGFα such as mucosal resistance to ethanol injury. In the present study, we investigated the long-term effects of TGFα on ethanol-induced gastric injury by analyzing changes in cytoprotective and anti-apoptotic regulatory factors, including gastric blood flow, NO synthase, COX-2, and apoptosis-associated proteins such as HSP70, Bcl-2, and caspase 3.

## Methods

### TGFα-expressing transgenic mouse and ethanol-induced gastric injury

TGFα-expressing transgenic (TG) mouse lines bearing human TGFα cDNA under the control of the mouse metallothionein gene I promoter were generated on a CD1 or FVB mouse background, as described previously [20]. One of the TG lines, MT100 on an FVB mouse background, displayed hypertrophic gastropathy resembling Menetrier's disease [20], whereas the other line, MT42 on a CD1 mouse background, displayed a normal-appearing gastric mucosa. The phenotype of the TGFα-TG mouse appeared to depend on the mouse strain, because both the MT100 and MT42 lines expressed a similar level of the TGFα transgene in the stomach [20]. In the present study, we used the MT42 line of TGFα-expressing TG male mice and CD1 wild type (WT) male mice at ages 6 to 8 weeks. The animals were maintained under controlled light (7:00 AM to 7:00 PM) with food and water provided *ad libitum*.

The mice, weighing 30–35 g, were fasted for 24 h before the experiment but were allowed free access to water. All experimental procedures affecting the mice were approved by the Animal Care and Use Review Committee at the Gunma University. The mice were then given orogastric acidified ethanol (100 μl; 60% ethanol in 0.15 mol/L HCl). Three, six, and twelve hours after the acidified ethanol administration the mice were sacrificed for the evaluation of gastric injury and immunohistochemical analyses, and for RNA and protein extraction for real time PCR, Northern and Western blot analyses.

### Physiological studies

Gastric acid secretion was measured as described previously [20,23]. Briefly, WT and TG mice were fasted for 3 h and then anesthetized with ether. After the incision of abdominal wall, the pyrolus was ligated, and the incision was sutured. The gastric fluid was collected 4 h after the pyrolus ligation. For maximal acid output, acid secretion was stimulated by injecting pentagastrin subcutaneously (500 μg/kg body weight). The gastric fluid was titrated with 0.1 N NaOH to pH 7.0 using a microtitrator.

Gastric mucosal blood flow was measured with a laser Doppler flowmeter (model SFA211, Advance Co., Tokyo) by placing a probe on the surface of the gastric mucosa, as described previously [24]. The flow signal was monitored with the MacLab recording program. The blood flow was expressed relative to the basal level.

### Morphological studies

The thickness of the gastric mucosa was measured from the highest part to the bottom of the gland with a micrometer. Similarly, the gastric injury lesions were measured (width and longitude) with a micrometer to calculate the injured area.

For immunostaining, we obtained antibodies as follows. Polyclonal antibody to H^+^/K^+^-ATPase was obtained by injecting rabbit parietal cell-microsomal fractions into mice [25]. Rabbit polyclonal antibody to metallothionein was a kind gift from Dr. Nagamine, Gunma University School of Health Sciences [26]. We purchased antibodies as follows: rabbit polyclonal antibody to TGFα, rabbit polyclonal antibody to EGF-R, goat polyclonal antibody to COX-2, rabbit polyclonal antibody to NFκB, and mouse monoclonal antibody to nNOS and iNOS from Santa Cruz Biotech. (Santa Cruz, CA); mouse monoclonal antibody to proliferative-cell nuclear antigen (PCNA) from Zymed Lab. (South San Francisco, CA); sheep antibody to human pepsinogen II from BioPur AB (Bubendorf, Switzerland), which also reacts to mouse pepsinogen; rabbit polyclonal antibody to HSP70 from Stressgen Biotech. (Victoria, BC, Canada); and mouse monoclonal antibody to Bcl-2 from BD Biosciences (San Diego, CA).

For horseradish peroxidase (HRP)-catalyzing immunohistochemical staining, minced stomachs were fixed in neutralized 10% formalin at 4°C for 24 h, and was then embedded in paraffin for microtome sectioning. The deparaffinized tissue sections were rehydrated and incubated in 3% hydrogen peroxide solution to inhibit endogenous peroxidase activity. Then the sections were incubated with a first antibody described above with an appropriate dilution at room temperature for 30 min. A secondary biotinylated antibody was selected based on the first antibody-producing animal species, and was then used with HRP-conjugated streptavidin. The HRP reaction was performed with 3-amino-9-ethyl-carbazole and hydrogen peroxide according to the instructions supplied with the streptavidin-biotin staining Vectastatin Elite Kit (Vectors Laboratories, Burlingame, CA). Positive stainings appeared brown in color.

For immunofluorescent staining, minced stomachs were fixed in 4% paraformaldehyde in 0.1 M phosphate buffer, pH 7.4 at 4°C for 24 h. Small pieces of minced tissue underwent saccharose replacement and were embedded in OCT-compound in preparation for a frozen section using a microtome. For the primary immunoreaction, sliced sections were probed with a first antibody, and were then incubated either with indodicarbocyanide (Cy3)-conjugated affinity-purified donkey anti-rabbit, anti-mouse IgG (Jackson ImmunoResearch, West Grove, PA), or fluorescein isothiocyanate (FITC)-labeled anti-rabbit IgG (Jackson ImmunoResearch), as a secondary antibody. The secondary antibody IgG was selected based on the species on which the first antibody was raised.

In situ detection of apoptotic cells was performed using the In Situ Apoptosis Detection Kit (TAKARA BIO INC. Tokyo, Japan). The kit contained a terminal deoxynucleotidyl transferase-mediated deoxyuridine triphosphate biotin nick end-labelling (TUNEL) assay system. Stomach sections were permeabilized with proteinase K, and the 3'-OH ends of the DNA fragments were then stained following the instructions supplied with the kit. The nuclei stained dark brown were considered to be apoptotic cells.

### RNA analysis

Stomachs were trimmed and the total RNAs were extracted using TRIzol (Gibco BRL, Tokyo) according to the protocol supplied by the manufacturer. For Northern blot, the total RNA was electrophoresed on a 1.0% agarose gel and was transferred to a nylon membrane (Amersham Pharmacia Biotech, Tokyo). Hybridization was performed with a probe of the human TGFα cDNA (917 bp) labeled with [α-^32^P] deoxy-CTP. This probe recognizes human TGFα mRNA, but does not mouse TGF mRNA.

Mouse TGFα mRNA level was measured using a real time polymerase chain reaction (PCR) with glyceraldehyde-3-phosphate dehydrogenase (GAPDH) as an internal control. The real time PCR was carried out using the ABI Prism 7700 Sequence Detector and soft (Applied Biosystems, Foster City, CA) using the DNA fluorescent dye SYBR Green detection. Primers were designed using the Primer Express design software (Applied Biosystems). The final result for each sample was normalized by the respective GAPDH value. Primer sequences are as follows: mouse TGFα: 5'-CCTGAGCACCCGAAGAT-3' and 5'-CCTTCCCTCATGCCTTACT-3'; GAPDH: 5'-GTCGTGGATCTGACGTGCC-3' and 5'-TGCCTGCTTCACCACCTTCT-3'.

### Western blot analysis

Gastric tissue samples were trimmed and total protein from the gastric mucosa was homogenized in RIPA buffer containing a cocktail of protease inhibitors (phenylmethylsulphonyl fluoride, pepstatin, leupeptin, and aprotinin) (Roche Diagnostics, Mannheim, Germany). The supernatant was separated on an SDS-PAGE gel, and was then blotted to a polyvinylidene difluoride membrane. The membrane was probed with the following antibodies: antibodies to iNOS, COX-1, COX-2, HSP70, and Bcl-2, which were the same ones used for morphological studies; mouse monoclonal antibody to Bax (Santa Cruz Biotech.); rabbit polyclonal antibody to caspase 3 (Santa Cruz Biotech.); and mouse monoclonal antibody to actin filaments (Santa Cruz Biotech.). Antibody-reacted bands were detected utilizing an ECL detection system (Amersham, Buckinghamshire, UK).

### Measurement of gastrin

Serum gastrin was measured using a gastrin radioimmunoassay kit (gastrin RIA kit, Dainabot, Tokyo, Japan). The antibody is specific for gastrin with an amide moiety at its carboxyl terminus.

### Measurement of Prostaglandin E_2_

The gastric mucosa was homogenized at 4°C in lysis buffer. Homogenates were centrifuged at 12000 rpm for 20 min, and the supernatants were subjected to prostaglandin E_2 _assay using a Prostaglandin E_2 _Monoclonal Enzyme Immunoassay Kit (Cayman Chemical, Ann Arbor, MI), according to the manufacturer's instruction.

### Statistical analysis

Statistical analysis was performed by repeated measure analysis of variance (ANOVA) for the total length of the gastric lesions by unpaired t-test for gastric mucosal blood flow decrease ratio. All values are expressed as the mean ± SE. P < 0.05 was accepted as statistically significant.

## Results

### Characterization of the TGFα-expressing TG mouse gastric mucosa

The WT CD1 strain and TGFα-transgene expressing MT42 line mice grew similarly and showed no noticeable difference in their features, behaviors, or lifespans. The greatest thickness observed for the gastric wall and fundic glands were 3.50 ± 0.18 mm and 2.33 ± 0.20 mm, respectively, in the WT mice, and 3.85 ± 0.24 mm and 2.63 ± 0.16 mm, respectively, in the TG mice; there were no significant differences in the shape of the gastric mucosae and glands between the WT and TG mice. In addition to the normal appearance of the gastric mucosa, this TG mouse line also had a normal-appearing liver, pancreas, and other organs.

**Figure 1:**
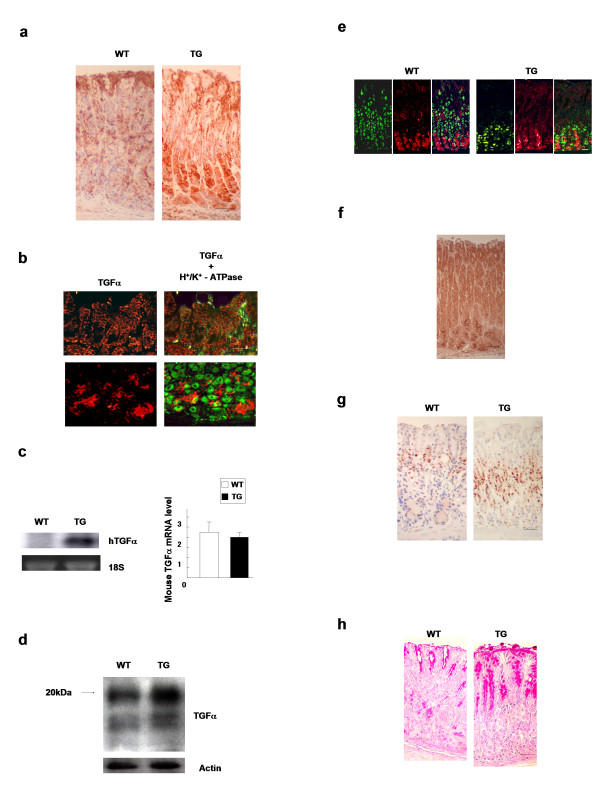
**Characterization of the TGFα-expressing TG mucosa**. Scale in each figure indicates 100 μm. (**a**) Distribution of TGFα-expressing cells. Brown-colored TGFα-positive cells were visible along the luminal pit region in the WT mucosa, the elongated foveolar pit region, and the lower one-third of the glandular region in the TG mucosa. (**b**) Overlap of red-colored TGFα-positive cells and green-colored H^+^/K^+^-ATPase-positive parietal cells. Lower TGFα-positive cells in cluster are distinct from the green-colored parietal cells in the TG mucosa. (**c**) Northern blot of human TGFα mRNA and PCR-amplified mouse TGFα mRNA. Human TGFα mRNA is expressed in the TG mucosa alone (left panel), but PCR-amplified mouse TGFα mRNA is expressed similarly in both WT and TG mucosae (right panel). (**d**) Western blot of TGFα intermediate forms. TGFα precursor (20 kDa) and its intermediate forms are visualized more intensively in the TG mucosa than in the WT mucosa. (**e**) Localization of EGF receptor in the gastric mucosa. EGF receptor (EGF-R) was stained with Cy3 (red) and H^+^/K^+^-ATPase was stained with FITC (green). EGF-R was immunostained moderately in the upper pit region and strongly in the lower glandular region similarly in both the WT and TG mucosae. (**f**) Distribution of metallothionein in the gastric mucosa. Metallothionein is immunostained strongly in the lower glandular region, and moderately along the foveolar region. (**g**) Elongation of the gastric pit of the TG mucosa. PCNA-positive cells are distributed at the upper-third region in the WT gastric glands, whereas they are distributed more numerously and broadly in the middle glandular region. (**h**) PAS staining shows elongated pit in the TG mucosa.

We then compared acid secretion capacity between the WT and TG mice. In the WT mice, maximal acid output increased significantly from basal level of 25 ± 1.4 mEq/h to 50 ± 5.8 mEq/h after the pentagastrin stimulation. By contrast, in the TG mice, maximal acid output increased to a less extent to 30 ± 5.7 mEq/h from the basal output of 23 ± 0.7 mEq/h (n = 5 in WT and TG mice). The blunted acid output may reflect the reduced parietal cell mass in the TG mucosa (Figure 1e).

### Expression of TGFα transgene

TGFα-expressing cell-types were identified based on cell-type-specific immunostaining and their mucosal location. TGFα was reportedly immunostained along the GSM cells in the gastric pit in humans [14] and rats [15], and along the mucous neck cell region in the FVB strain mice [16]. In the present study, brown-colored TGFα-positive cells were distributed along the foveolar region of both the WT and TG mucosae, and along the lower glandular region of the TG mucosa (Figure 1a). The TGFα-positive cells along the foveolar region appeared to be gastric pit cells, based by their location (Figure 1b, upper panel), and those along the lower glandular region of the TG mucosa did not overlap with the green-colored H^+^/K^+^-ATPase-positive parietal cells (Figure 1b, lower panel), but did overlap with the pepsinogen-positive chief cells (data not shown). The antibody we used for immunostaining for TGFα could not distinguish between human and mouse TGFα, but its mRNA probe could do so for the Northern blot analysis. The human TGFα-transgene was expressed in the TG mucosa while no expression was noted in the WT mucosa (Figure 1c, left panel). In contrast, endogenous mouse TGFα mRNA was similarly expressed in both the WT and TG mucosae by real time PCR (Figure 1c, right panel). TGFα is a 50 amino acid peptide, derived from its 160 amino acid membrane-spanning precursor cleaved by tumor necrosis factor-α-converting enzyme (TACE) [27,28]. With the surplus expression of the human TGFα-transgene in the TG mucosa, an approximately 20 kDa TGFα precursor and its intermediate forms were increased by immunoblotting (Figure 1d).

Since both the precursor and processed forms were equally active for stimulating EGF-R [28], we further examined EGF-R localization in the mucosa. EGF-R was localized heavily in the lower chief cell cluster region and moderately in the mucous neck cell region and in the foveolar pit region in both the WT and TG mucosae (Figure 1e), as was reported previously for the rat gastric mucosa [15]. Since the TGFα transgene was controlled under the mouse metallothionein gene [20] and metallothionein has been reportedly produced in the gastric mucosa [29], we examined the metallothionein-expressing cell-types in the mucosa. Immunohistochemically, we noted metallothionein staining mostly along the lower glandular region and scatteringly along the pit region of the control CD1-strain gastric mucosa (Figure 1f). Thus, the distribution of TGFα-expressing cell-types is similar to that of metallothionein-expressing cells in the TG gastric mucosa (Figures 1a and 1f).

In the WT mouse mucosa, the H^+^/K^+^-ATPase-positive parietal cells were spread widely over the glandular region except for the top-pit layer, whereas the H^+^/K^+^-ATPase-positive glandular region was reduced in height in the TG mucosa (Figure 1e). Indeed, the isthmus at the base of the pit region, which was confirmed by PCNA staining, was moved down to the middle of the TG mucosa (Figure 1g). Moreover, PCNA-positive cells were thickly distributed in the middle of the mucosa. Consistently, the PAS-staining-positive foveolar pit was elongated in the TG mucosa in contrast to the short pit in the WT mucosa (Figure 1h). Thus, although the height of the gastric mucosa was similar between the WT and TG mucosae, the proliferative zone was moved down and the foveolar pit region was more elongated with a reduced glandular region in the TG mocosa. Since the growth of pit cells is strongly enhanced by gastrin [25,30], we compared the serum gastrin levels between the WT and TG mice but found no increase in the levels of serum gastrin in the TG mouse (WT, 213 ± 41 pg/ml; TG, 263 ± 47 pg/ml, n = 5, p = 0.438).

### Ethanol-induced gastric injury in the WT and TG mouse mucosae

**Figure 2:**
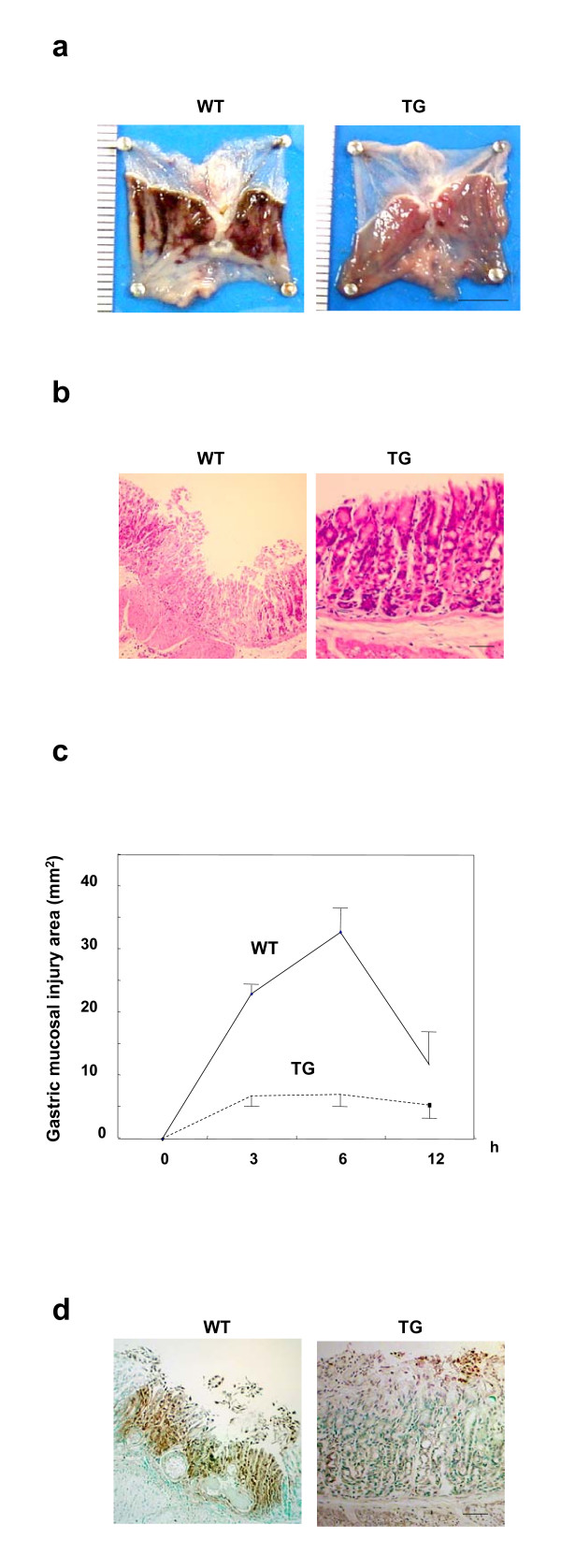
**Ethanol-induced gastric injury in the gastric mucosae**. (**a**) Macroscopic view of the gastric mucosa. Scale: 5 mm. Both mucosae are 6 h after ethanol administration. Note that the WT mucosa displays extensive hemorrhagic lesions, in contrast, the TG mucosa looks undamaged after ethanol administration. (**b**) Hematoxylin and eosin staining of ethanol-injured WT mucosa (left) and TG mucosa (right). Note the deep ulcer formation in the WT mucosa. Scale: 100 μm. (**c**) Time course of ethanol-induced gastric injury. Injured area is plotted against time up to 12 h after the ethanol administration. Each point represents an average of five mice. *p *< 0.05 (**d**) TUNEL staining of WT mucosa (left) and TG mucosa (right). Both sections are consecutive to those in 2*B*, respectively. Apoptotic cells are visible with dark-brown nuclei. Numerous apoptotic cells are noted in the WT mucosa. All photos were 6 h after the ethanol administration. Scale: 100 μm.

Orogastric administration of acidified ethanol (100 μl) caused hemorrhagic injury in the TG mucosa (Figure 2a, left). Histologically the injury peeled off the pit and isthmus regions and reached to the mid-glandular region (Figure 2b, left). In contrast, this sort of injury in the TG mouse mucosa was not notable (Figure 2a, right). The injury was histologically limited to the luminal pit region (Figure 2b, right). In the WT mucosa, the injured area increased rapidly with a peak at 6 h after the ethanol administration, whereas in the TG mucosa the area increased slowly and minimally without a marked peak (Figure 2c). Consistently, apoptotic cells shown by the TUNEL staining distributed deeply to the lower glandular region 3 h after the ethanol administration in the WT mucosa, whereas the apoptotic cells were confined to the pit region but did not extend to the glandular region in the TG mucosa (Figure 2d).

### Gastric mucosal blood flow in the ethanol-treated mucosa

**Figure 3:**
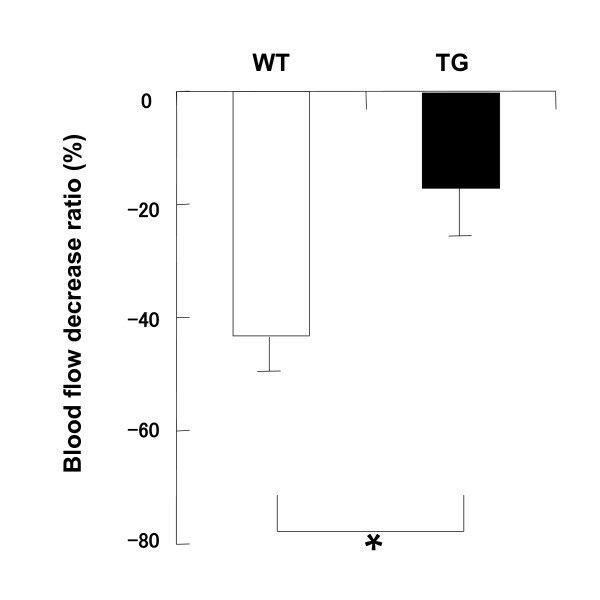
**Assessment of gastric mucosal blood flow in the gastric mucosae**. Gastric mucosal blood flow was assessed by comparing the blood flow ratio before and after the ethanol treatment. The ratio was obtained as a percent against the blood flow before the treatment, which was much larger in the WT mucosa than in the TG mucosa. *p < 0.03.

Although a number of cytoprotective regulatory factors have been reported, including NO, CGRP, COX2, and HSP70 [12,13,31,32], mucosal blood flow has been proposed as a primary factor for the protection and restitution of ethanol-induced gastric injury [1,33]. We measured gastric blood flow by placing a probe of the laser Doppler flowmeter on the gastric mucosa accessed via an exposed abdominal wall in the anesthetized mouse. Gastric blood flow decreased marked by 43.5 ± 6.37% after the ethanol administration on the gastric mucosa of the WT mouse (Figure 3). In contrast, it only decreased by 17.2 ± 9.21% after the ethanol administration on the TG mucosa (Figure 3). Thus, the gastric blood flow was well maintained in the TG mucosa even after ethanol treatment. Since mucosal ischemia is one of the most important ulcerogenic factors [33], the maintenance of gastric blood flow does appear to be an essential factor for mucosal protection.

### Expression of cytoprotective proteins: NO synthase, COX-2, and HSP70

**Figure 4:**
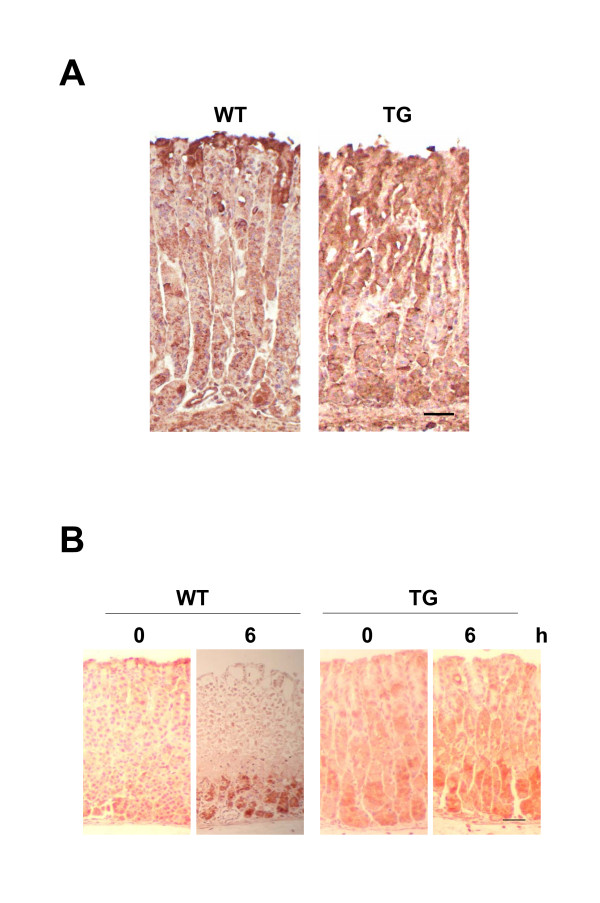
**Immunostaining of NO synthases in the gastric mucosa**. Scale: 100 μm. (**a**) nNOS immunostaining. nNOS was visible along the pit region, resulting in its wider distribution along the elongated pit in the TG mucosa. (**b**) iNOS immunostaining. iNOS was induced in the lower glandular region after ethanol injury in the WT mucosa, whereas it was constitutively expressed in the same region of the TG mucosa irrespective of ethanol injury.

To explore the mechanism of high blood flow maintenance in the TG mucosa, we examined the expression of NO synthases, which makes NO from arginine, and relaxes vascular smooth muscles via cGMP-dependent protein kinase [34]. There are three types of NO synthase (NOS): neural NOS (nNOS), inducible NOS (iNOS), and endothelial NOS (eNOS). nNOS and eNOS are constitutively expressed, whereas iNOS is induced upon gastric injury [35]. nNOS was distributed similarly to the PAS-staining over the foveolar pit region (Figure 1g); it was observed over the elongated pit of the TG mucosa and was limited to the luminal pit of the WT mucosa (Figure 4a). eNOS was weakly scattered over the entire gastric mucosa similarly in both the WT and TG mucosae (data not shown). The iNOS staining was negligible in the WT mucosa, although it was highly induced in the lower glandular region after the mucosa was exposed to ethanol (Figure 4b). In the TG mucosa, iNOS was constitutively expressed in the lower glandular region similarly before and after the injury (Figure 4b), which appears to further contribute to the maintenance of high blood flow.

**Figure 5:**
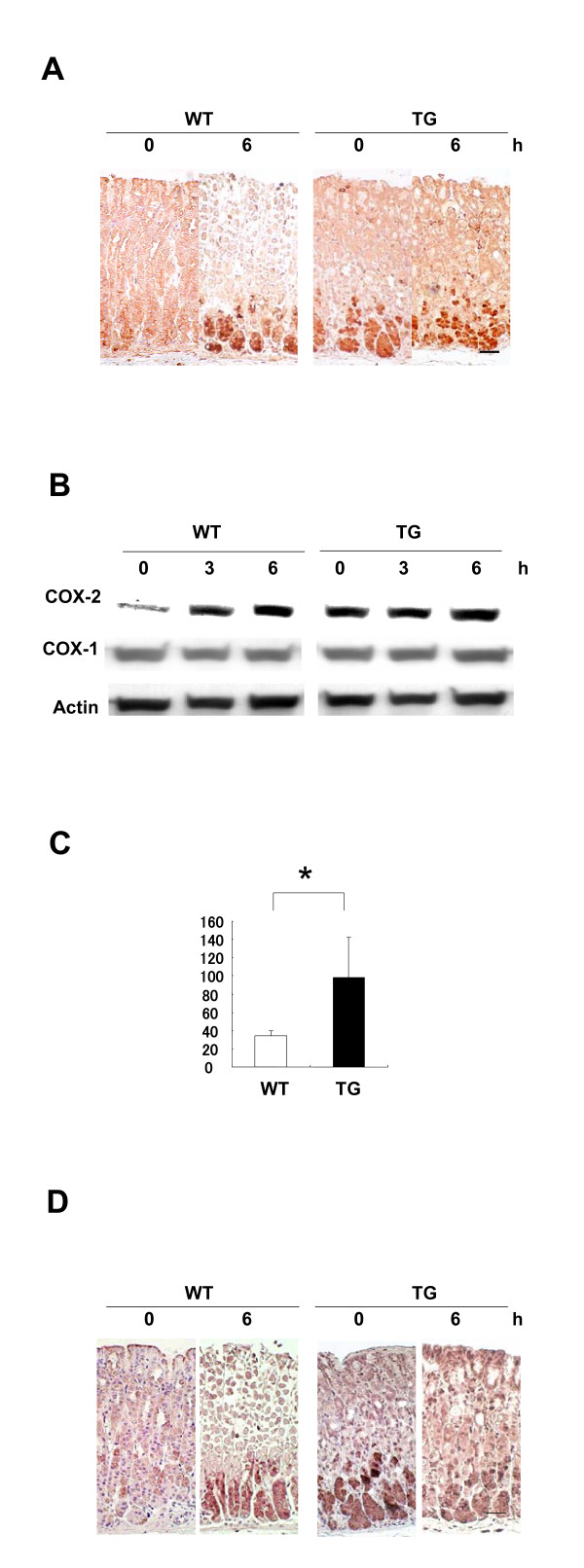
**COX-2 and NFκB expression in the gastric mucosa**. (**a**) Immunostaining of COX-2. COX-2 was intensively induced after the injury at the lower glandular region of the WT mucosa, whereas it was constitutively expressed in the same region of the TG mucosa even before the injury. Ethanol-injured mucosa was stained 6 h after the ethanol administration. Scale: 100 μm. (**b**) Immunoblotting of COX-2 and COX-1. The blots were measured 0, 3, and 6 h after the injury. Actin filaments were used as a control. Note that COX-2 was intensively expressed before the injury in the TG mucosa. (**c**) Measurement of prostaglandin E_2_. Prostaglandin E_2 _levels were measured before the ethanol treatment in the WT and TG mucosae. n = 5, p = 0.013. (**d**) Immunostaining of NFκB. NFκB was intensively induced after the injury at the same lower glandular region of the WT mucosa as was COX-2. In contrast, NFκB was constitutively expressed in the same region of the TG mucosa even before the injury. Scale: 100 μm.

Prostaglandins were proposed as a major cytoprotective factor over three decades ago [8] and their roles have been extensively studied [36,37]. The role of the prostaglandin-forming enzymes, COX-1 and COX-2, has been controversial in the mucosal protection against gastric irritants [7,11,32]. We examined the expression of COX in the WT and TG mucosae during ethanol injury. The expression of COX-1 did not differ between the WT and TG mucosae, nor before or after the ethanol injury (Figure 5b). COX-2 was not detectable in the WT mucosa, but its expression became evident 6 h after ethanol injury, and its staining was localized to the lower glandular region (Figure 5a and 5b). In the TG mucosa, the COX-2 immunostaining was intense at the same region even before the injury and remained positive after the injury (Figure 5a). Since COXs have been shown to be expressed in mesenchymal cells such as fibroblasts and mononuclear cells in the gastric mucosa [38], and the streptoavidin/biotin/HPR method sometimes presents false-positive staining, we performed immunofluorescent staining using a frozen section which confirmed COX expression similarly in the lower glandular region of the TG mucosa (data not shown). By immunoblotting, COX-2 increased after the injury in the WT mucosa, whereas it was highly elevated to a similar extent before and after the injury (Figure 5b). Consistently, prostaglandin E_2 _levels were significantly higher in the TG mucosa than in the WT mucosa (Figure 5c). Furthermore, NFκB, a transcription factor responsible for COX-2 gene expression, was localized to the lower glandular region of the TG mucosa even before the ethanol treatment (Figure 5d), suggesting that TGFα transgene causes the constitutive expression of NFκB [21].

**Figure 6:**
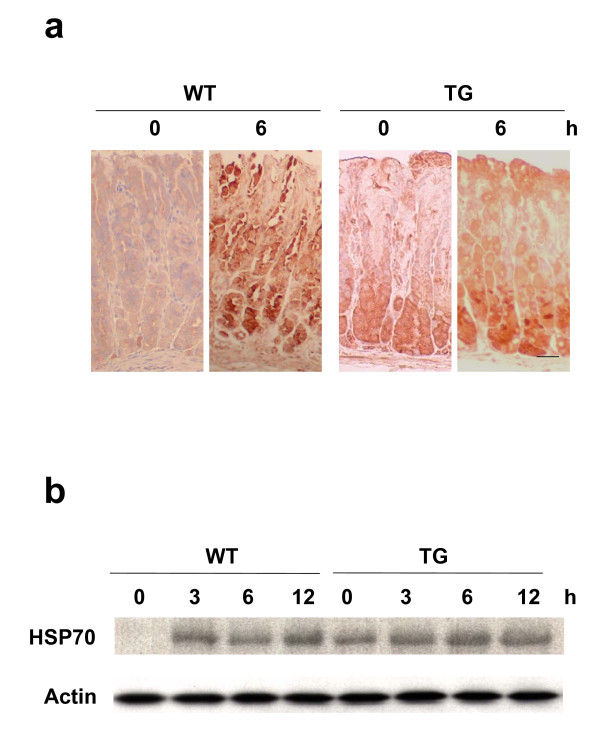
**HSP70 expression in the gastric mucosa**. (**a**) Immunostaining of HSP70. HSP70 was induced along the necrotic region and the lower glandular region of the WT mucosa after ethanol injury, whereas it was constitutively expressed in the lower glandular region of the TG mucosa before and after the injury. Ethanol-injured mucosa was stained 6 h after the ethanol administration. Scale: 100 μm. (**b**) Immunoblotting of HSP70. HSP70 blot was assessed 0, 3, 6, 12 h after the ethanol injury. HSP was induced after ethanol injury in the WT mucosa, while it was constitutively expressed in the TG mucosa before and after the injury.

Gastric irritants are known to induce heat shock protein 70 (HSP70), which chaperones denatured proteins for renaturation or clearance [39]. HSP70 was not detected in the WT mucosa, but after ethanol injury it was observed extensively over the glandular region, and scatteringly in the pit region of the WT mucosa (Figure 6a). In contrast, it was visible at the lower glandular region of the TG mucosa similarly before and after ethanol treatment (Figure 6a). By immunoblotting, HSP70 was not detected before ethanol injury but appeared 3 h to 12 h after the injury in the WT mucosa (Figure 6b). In contrast, it was already elevated before the injury and increased moderately after the injury (Figure 6b).

Collectively, the three cytoprotection-associated proteins, iNOS, COX-2, and HSP70, were negligible in expression before the injury and highly induced after the injury in the WT mucosa, but they stayed elevated similarly before and after the injury in the TG mucosa.

### Expression of apoptosis-associated proteins in the gastric mucosa

**Figure 7:**
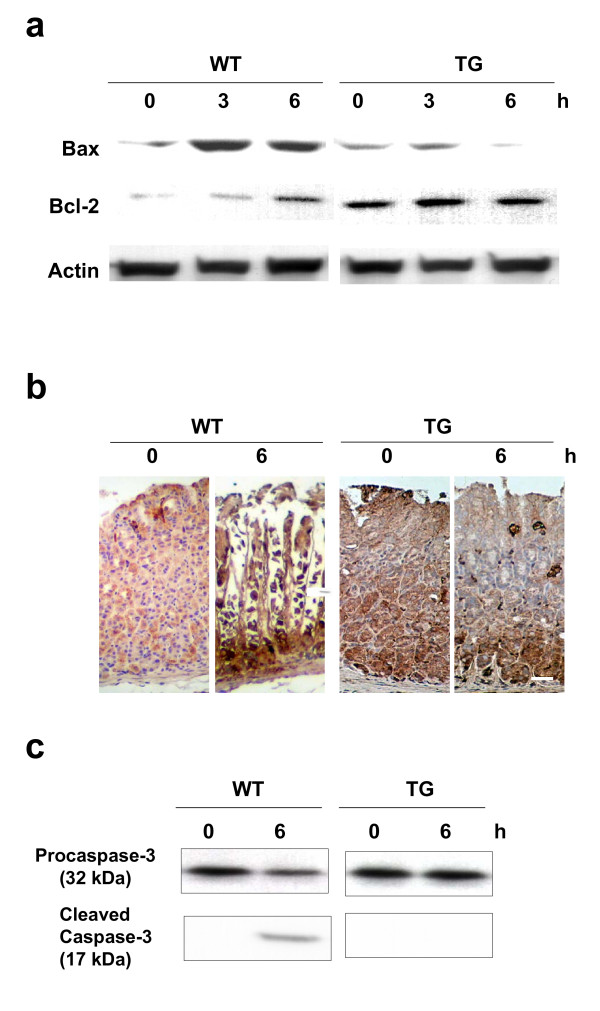
**Expression of apoptosis-associated proteins**. (**a**) Immunoblot of Bax and Bcl-2. Bax was strongly induced at the 3 h and 6 h time points in the WT mucosa, whereas it was reduced to a negligible level in the TG mucosa after ethanol injury. Bcl-2 was stably expressed similarly before and after the injury in the TG mucosa, whereas it was increased to some extent at 6 h after the injury in the WT mucosa. (**b**) Immunostaining of Bcl-2. Bcl-2 was not visible in the TG mucosa before the injury, but appeared along the border region of necrotic area after the injury. In contrast, Bcl-2 was constitutively induced over the TG mucosa even before the injury, and the staining was confined to the lower glandular region after the injury. Scale: 100 μm. (**c**) Immunoblot of caspase 3. After the injury, a 17 kDa active form of caspase 3 appeared in the WT mucosa, whereas it did not in the TG mucosa. A 32 kDa proform was unchanged both in the WT and TG mucosae.

As shown with the TUNEL staining in Figure 2d, the TGFα-expressing mucosa was strongly resistant to ethanol injury. Initially, we examined the expression of anti-apoptotic Bcl-2 and pro-apoptotic Bax by immunoblotting. Bcl-2 and Bax were slightly expressed in the WT mucosa before the injury; after the injury, Bax increased intensively whereas Bcl-2 increased to a lesser extent (Figure 7a). In the TG mucosa, Bcl-2 was expressed even before the injury and remained elevated after the injury, whereas Bax decreased to a negligible level after the injury. By immunostaining, Bcl-2 was positive in the lower glandular region of the TG mucosa before the injury, but no staining was visible in the WT mucosa. After the ethanol injury, Bcl-2 appeared strongly along the periphery of the injured lesion in the WT mucosa, in contrast, it stained similarly to the pretreatment level in the TG mucosa (Figure 7b). Thus, the constitutive expression of Bcl-2 may contribute to the anti-apoptotic feature of the TG mucosa against ethanol injury. Evidence of the active form of caspase-3, an executive caspase, was consistently absent in the TG mucosal cell extract before and after the injury, whereas it was intensively detected in the WT mucosa after the injury, although the proform remained similarly before and after the injury in both the WT and TG mucosae (Figure 7c).

## Discussion

EGF family growth factors including TGFa have been proposed as integrative cytoprotective factors against gastric injury [2,4]. The effects of TGFa have been tested by administrating it to animals or culture cells for a short term, and thus the reported cytoprotective mechanisms of TGFa have been somewhat variable [7,12,24,31,40-42]. TGFa is known to be a potent growth-promoting factor leading to hypertrophic growth in the gastric mucosae [19,20]. In contrast, the EGF-R signal is known to activate non-proliferative functions such as the induction of potent cytoprotective trefoil peptides, which are produced from the gastrointestinal mucosa, through an EGF-R/MAP kinase pathway [43]. Therefore, although high levels of TGFa can cause hypertrophic gastropathy, appropriate levels appear to maintain mucosal resistance to gastric injury. However, since the broad presence of PCNA-positive cells shown in Figure 1g indicates the proliferative action of TGFa to gastric precursor cells in the TG mucosa, it does not seem appropriate to categorize the action of TGFa as either proliferative or non-proliferative for gastric protection. More appropriate is to evaluate the diverse effects of TGFa using a long-term TGFa-expressing model. Using such a TGFa-transgene-expressing TG mouse model whose gastric mucosa showed a strong resistance to ethanol injury (Figure 2), we found that the integrative mechanisms of TGFa involve at least three categories of cytoprotective regulators: NO synthases in relation with gastric blood flow, COX-2, and apoptosis-associated regulators such as Bcl-2 and Bax.

Gastric blood flow is known to contribute substantially to mucosal protection against gastric injury [33]. In the present study, a laser Doppler flowmeter analysis revealed a significant maintenance of blood flow when acidified ethanol was applied onto the mucosal surface of the TG mouse (Figure 3). Since vascular dilation occurs even in the high dose capsaicin-denervated gastric mucosa where increased levels of NO contribute greatly [13], we confirmed the expression of NOS in the mucosa. nNOS was expressed at the luminal pit region as previously reported [35]. Since the pit region is elongated to half of the gastric gland in length in the TG mucosa, the nNOS-expressing region was much wider than that in the WT mucosa. Furthermore, iNOS was constitutively expressed along the lower glandular region in the TG mucosa, in contrast to the negligible expression of iNOS unless ethanol was applied to the WT mucosa (Figure 4). nNOS has been reported to be induced by EGF in guinea pig gastric mucosal primary-cultured cells [44], and in human HaCaT keratinocytes [45]. In contrast, iNOS, a key regulator of oxidant-induced epithelial barrier disruption, has been reportedly downregulated through an EGF-R downstream ζ-isoform of protein kinase C for gastric protection using colorectal carcinoma-derived Caco-2 cells [46]. We postulate that the long-term effect of TGFα on iNOS induction may be different from its short-term effect seen in culture cells.

Previously, prostaglandins have been shown to increase in parallel with the expression of COX-2 protein after ulcer formation in rats [32]. However, intraperitoneal administration of TGFα did not induce prostaglandin E_2 _release into the gastric juice nor did it increase gastric tissue levels of prostaglandin E_2 _[7]. The protective effect of TGFα to ethanol-induced mucosal injury was not interrupted by indomethacin-induced depletion of prostaglandin E_2 _in the rat gastric mucosa [42]. Although the roles of prostaglandins in gastric protection are controvertial, we found that COX-2 and its upstream transcription factor NFκB are markedly expressed in the TGFα-expressing TG mucosa (Figure 5a and 5d). Our findings suggest that COX-2 is induced by NFκB under the control of TGFα in gastric mucosal cells. Consistently, prostaglandin E_2 _levels were muchi more elevated in the TG mucosa than in the WT mucosa (Figure 5c).

In response to environmental or physiological stress, such as exposure to heat, ethanol, heavy metals, and water-immersion stress, heat shock proteins such as HSP70 are induced in the gastric mucosa [39,47]. HSPs are known to play a role in refolding of denatured proteins and facilitate the recovery of damaged cellular functions. HSP70 is also thought to interrupt stress-induced apoptosis pathways at several steps, including a cytochrome C releasing step from mitochondria, and a pro-caspase activation step by directly interacting with Apaf-1 [48]. In the present study, HSP70 was already elevated in the TG mucosa before ethanol injury, in contrast to the almost completely lack of HSP70 expression without injury in the WT mucosa (Figure 6). Together with HSP70, anti-apoptotic Bcl-2 remained similarly elevated before and after the ethanol injury, but pro-apoptotic Bax was down-regulated in the TG mucosa after the injury (Figure 7). Bcl-2 is an anti-apoptotic protein which resides on the mitochondrial outer membrane and inhibits channel formation of Bax, a pro-apoptotic protein of the same Bcl-2 family, through which cytochrome C leaks to the cytoplasm [49,50]. Thus, constitutive expression of Bcl-2 by TGFα may overcome unfavorable ethanol-exposure stress at least by suppressing Bax activation. Once the Bcl-2 function is suppressed, cytochrome C and other apoptogenic factors are released through the Bax channel, and cytochrome C associates with Apaf-1 to promote the activation of the initiator caspase, pro-caspase 9, whose active form subsequently activates the effecter caspase, pro-caspase 3. Caspase 3 cleaves the inhibitor of caspase-activated DNase (ICAD) to release CAD, which then causes DNA fragmentation [50,51]. In the present study, active 17 kDa caspase 3 was not formed in the TG mucosa, whereas it was markedly formed in the WT mucosa after the injury. Thus, we propose that the inhibitory effect of Bcl-2 on the release of cytochrome C, and the HSP70 block of cytochrome C-initiated apoptosome formation to produce activated caspase 9, resulted in the suppression of caspase 3 formation from its precursor [50,52].

The strong resistance of TGFα-expressing gastric mucosa to ethanol injury appears to involve at least three cytoprotective mechanisms: a well-maintained gastric blood flow partly due to NO synthase expression by TGFα; constitutive expression of COX-2, which produces prostaglandins that may recruit GSM precursor cells to the injured pit region [53]; and upregulation of anti-apoptotic proteins such as HSP70 and Bcl-2, downregulation of pro-apoptotic Bax, and inhibition of pro-caspase 3 activation. These distinct cytoprotection mechanisms appear to connect in an integrated manner.

## Conclusion

We propose that non-pathological levels of long-term TGFα expression may enhance the protective capacity of the gastric mucosa against injury and may help maintain the integration of mucosal functions by activating other essential cytoprotective factors such as mucosal blood flow, COX-2, and anti-apoptotic regulators such as HSP70 and Bcl-2 through these interconnecting pathways.

## Abbreviations

TGFα, transforming growth factor α; TG, transgenic; WT, wild-type; EGF-R, epidermal growth factor receptor; COX, cyclooxygenase: NOS, nitric oxide synthase; HSP, heat shock protein; GSM, gastric surface mucosa; HRP, horseradish peroxydase

## Competing interests

The author(s) declare that they have no competing interests.

## Authors' contributions

TK performed immunohistochemistry, Western blotting, Northern blotting, maintained transgenic mice, and performed genotype analysis with some help from SK, NS, NH and KS. MY performed gastric blood flow measurements. HT, TT and MM supervised the first author TK. All authors read and approved the final manuscript.

## Pre-publication history

The pre-publication history for this paper can be accessed here:



## References

[B1] Gonul B, Akbulut KG, Ozer C, Yetkin G, Celebi N (2004). The role of transforming growth factor alpha formulation on aspirin-induced ulcer healing and oxidant stress in the gastric mucosa. Surg Today.

[B2] Barnard JA, Beauchamp RD, Russell WE, Dubois RN, Coffey RJ (1995). Epidermal growth factor-related peptides and their relevance to gastrointestinal pathophysiology. Gastroenterology.

[B3] Kamimura H, Konda Y, Yokota H, Takenoshita S, Nagamachi Y, Kuwano H, Takeuchi T (1999). Kex2 family endoprotease furin is expressed specifically in pit-region parietal cells of the rat gastric mucosa. Am J Physiol.

[B4] Nakajima T, Konda Y, Izumi Y, Kanai M, Hayashi N, Chiba T, Takeuchi T (2002). Gastrin stimulates the growth of gastric pit cell precursors by inducing its own receptors. Am J Physiol Gastrointest Liver Physiol.

[B5] Potter CL, Hanson PJ (2000). Exogenous nitric oxide inhibits apoptosis in guinea pig gastric mucous cells. Gut.

[B6] Robert A, Nezamis JE, Lancaster C, Hanchar AJ (1979). Cytoprotection by prostaglandins in rats. Prevention of gastric necrosis produced by alcohol, HCl, NaOH, hypertonic NaCl, and thermal injury. Gastroenterology.

[B7] Slice LW, Hodikian R, Zhukova E (2003). Gastrin and EGF synergistically induce cyclooxygenase-2 expression in Swiss 3T3 fibroblasts that express the CCK2 receptor. J Cell Physiol.

[B8] Romano M, Polk WH, Awad JA, Arteaga CL, Nanney LB, Wargovich MJ, Kraus ER, Boland CR, Coffey RJ (1992). Transforming growth factor alpha protection against drug-induced injury to the rat gastric mucosa in vivo. J Clin Invest.

[B9] Sugiyama N, Tabuchi Y, Horiuchi T, Obinata M, Furusawa M (1993). Establishment of gastric surface mucous cell lines from transgenic mice harboring temperature-sensitive simian virus 40 large T-antigen gene. Exp Cell Res.

[B10] Sunnarborg SW, Hinkle CL, Stevenson M, Russell WE, Raska CS, Peschon JJ, Castner BJ, Gerhart MJ, Paxton RJ, Black RA, Lee DC (2002). Tumor necrosis factor-alpha converting enzyme (TACE) regulates epidermal growth factor receptor ligand availability. J Biol Chem.

[B11] Konda Y, Yokota H, Kayo T, Horiuchi T, Sugiyama N, Tanaka S, Takata K, Takeuchi T (1997). Proprotein-processing endoprotease furin controls the growth and differentiation of gastric surface mucous cells. J Clin Invest.

[B12] Karam S, Leblond CP (1995). Origin and migratory pathways of the eleven epithelial cell types present in the body of the mouse stomach. Microsc Res Tech.

[B13] Massague J (1990). Transforming growth factor-alpha. A model for membrane-anchored growth factors. J Biol Chem.

[B14] Orsini B, Calabro A, Milani S, Grappone C, Herbst H, Surrenti C (1993). Localization of epidermal growth factor/transforming growth factor-alpha receptor in the human gastric mucosa. An immunohistochemical and in situ hybridization study. Virchows Arch A Pathol Anat Histopathol.

[B15] Kang JY, Teng CH, Chen FC, Wee A (1998). Role of capsaicin sensitive nerves in epidermal growth factor effects on gastric mucosal injury and blood flow. Gut.

[B16] Takano H, Satoh M, Shimada A, Sagai M, Yoshikawa T, Tohyama C (2000). Cytoprotection by metallothionein against gastroduodenal mucosal injury caused by ethanol in mice. Lab Invest.

[B17] Peskar BM (2001). Neural aspects of prostaglandin involvement in gastric mucosal defense. J Physiol Pharmacol.

[B18] Chen MC, Lee AT, Soll AH (1991). Mitogenic response of canine fundic epithelial cells in short-term culture to transforming growth factor alpha and insulinlike growth factor I. J Clin Invest.

[B19] Dempsey PJ, Goldenring JR, Soroka CJ, Modlin IM, McClure RW, Lind CD, Ahlquist DA, Pittelkow MR, Lee DC, Sandgren EP (1992). Possible role of transforming growth factor alpha in the pathogenesis of Menetrier's disease: supportive evidence form humans and transgenic mice. Gastroenterology.

[B20] Takahashi S, Shigeta J, Inoue H, Tanabe T, Okabe S (1998). Localization of cyclooxygenase-2 and regulation of its mRNA expression in gastric ulcers in rats. Am J Physiol Gastrointest Liver Physiol.

[B21] Karam SM, Leblond CP (1993). Dynamics of epithelial cells in the corpus of the mouse stomach. I. Identification of proliferative cell types and pinpointing of the stem cell. Anat Rec.

[B22] Chen MC, Solomon TE, Kui R, Soll AH (2002). Apical EGF receptors regulate epithelial barrier to gastric acid: endogenous TGF-alpha is an essential facilitator. Am J Physiol Gastrointest Liver Physiol.

[B23] Li Z, Jansen M, Ogburn K, Salvatierra L, Hunter L, Mathew S, Figueiredo-Pereira ME (2004). Neurotoxic prostaglandin J2 enhances cyclooxygenase-2 expression in neuronal cells through the p38MAPK pathway: a death wish?. J Neurosci Res.

[B24] Zhang B, Hosaka M, Sawada Y, Torii S, Mizutani S, Ogata M, Izumi T, Takeuchi T (2003). Parathyroid hormone-related protein induces insulin expression through activation of MAP kinase-specific phosphatase-1 that dephosphorylates c-Jun NH2-terminal kinase in pancreatic beta-cells. Diabetes.

[B25] Konturek PC, Brzozowski T, Kania J, Konturek SJ, Hahn EG (2003). Nitric oxide-releasing aspirin protects gastric mucosa against ethanol damage in rats with functional ablation of sensory nerves. Inflamm Res.

[B26] Takagi H, Jhappan C, Sharp R, Merlino G (1992). Hypertrophic gastropathy resembling Menetrier's disease in transgenic mice overexpressing transforming growth factor alpha in the stomach. J Clin Invest.

[B27] Milani S, Calabro A (2001). Role of growth factors and their receptors in gastric ulcer healing. Microsc Res Tech.

[B28] Takagi H, Fukusato T, Kawaharada U, Kuboyama S, Merlino G, Tsutsumi Y (1997). Histochemical analysis of hyperplastic stomach of TGF-alpha transgenic mice. Dig Dis Sci.

[B29] Taupin D, Wu DC, Jeon WK, Devaney K, Wang TC, Podolsky DK (1999). The trefoil gene family are coordinately expressed immediate-early genes: EGF receptor- and MAP kinase-dependent interregulation. J Clin Invest.

[B30] Oshima M, Dinchuk JE, Kargman SL, Oshima H, Hancock B, Kwong E, Trzaskos JM, Evans JF, Taketo MM (1996). Suppression of intestinal polyposis in Apc delta716 knockout mice by inhibition of cyclooxygenase 2 (COX-2). Cell.

[B31] Mizuno H, Sakamoto C, Matsuda K, Wada K, Uchida T, Noguchi H, Akamatsu T, Kasuga M (1997). Induction of cyclooxygenase 2 in gastric mucosal lesions and its inhibition by the specific antagonist delays healing in mice. Gastroenterology.

[B32] Nasim MM, Thomas DM, Alison MR, Filipe MI (1992). Transforming growth factor alpha expression in normal gastric mucosa, intestinal metaplasia, dysplasia and gastric carcinoma – an immunohistochemical study. Histopathology.

[B33] Vongthavaravat V, Mesiya S, Saymeh L, Xia Y, Harty RF (2003). Mechanisms of transforming growth factor-alpha (TGF-alpha) induced gastroprotection against ethanol in the rat: roles of sensory neurons, sensory neuropeptides, and prostaglandins. Dig Dis Sci.

[B34] Rokutan K, Teshima S, Kawai T, Kawahara T, Kusumoro K, Mizushima T, Kishi K (2000). Geranylgeranylacetone stimulates mucin synthesis in cultured guinea pig gastric pit cells by inducing a neuronal nitric oxide synthase. J Gastroenterol.

[B35] Hirose M, Miwa H, Kobayashi O, Oshida K, Misawa H, Kurosawa A, Watanabe S, Sato N (2002). Inhibition of proliferation of gastric epithelial cells by a cyclooxygenase 2 inhibitor, JTE522, is also mediated by a PGE2-independent pathway. Aliment Pharmacol Ther 16 Suppl.

[B36] Helmer KS, West SD, Shipley GL, Chang L, Cui Y, Mailman D, Mercer DW (2002). Gastric nitric oxide synthase expression during endotoxemia: implications in mucosal defense in rats. Gastroenterology.

[B37] Yanaka A, Suzuki H, Shibahara T, Matsui H, Nakahara A, Tanaka N (2002). EGF promotes gastric mucosal restitution by activating Na(+)/H(+) exchange of epithelial cells. Am J Physiol Gastrointest Liver Physiol.

[B38] Polk WH, Dempsey PJ, Russell WE, Brown PI, Beauchamp RD, Barnard JA, Coffey RJ (1992). Increased production of transforming growth factor alpha following acute gastric injury. Gastroenterology.

[B39] Wallace JL (2001). Nonsteroidal anti-inflammatory drugs and the gastrointestinal tract. Mechanisms of protection and healing: current knowledge and future research. Am J Med.

[B40] Halter F, Tarnawski AS, Schmassmann A, Peskar BM (2001). Cyclooxygenase 2-implications on maintenance of gastric mucosal integrity and ulcer healing: controversial issues and perspectives. Gut.

[B41] Kanai M, Konda Y, Nakajima T, Izumi Y, Takeuchi T, Chiba T (2001). TGF-alpha inhibits apoptosis of murine gastric pit cells through an NF-kappaB-dependent pathway. Gastroenterology.

[B42] Watanabe D, Otaka M, Mikami K-I, Yoneyama K, Goto T, Miura K, Ohshima S, Lin J-G, Shibuya T, Segawa D, Kataoka E, Konishi N, Odashima M, Sugawara M, Watanabe S (2004). Expression of a 72-kDa heat shock protein, and its cytoprotective function, in gastric mucosa in cirrhotic rats. J Gastroenterol.

[B43] Tsukimi Y, Okabe S (2001). Recent advances in gastrointestinal pathophysiology: role of heat shock proteins in mucosal defense and ulcer healing. Biol Pharm Bull.

[B44] Sasaki E, Tominaga K, Watanabe T, Fujiwara Y, Oshitani N, Matsumoto T, Higuchi K, Tarnawski AS, Arakawa T (2003). COX-2 is essential for EGF induction of cell proliferation in gastric RGM1 cells. Dig Dis Sci.

[B45] Boissel J-P, Ohly D, Bros M, Godtel-Armbrust U, Forstermann U, Frank S (2004). The neuronal nitric oxide synthase is upregulated in mouse skin repair and in response to epidermal growth factor in human HaCaT keratinocytes. J INVEST DERMATOL.

[B46] Banan A, Zhang L, Fields JZ, Farhadi A, Talmage DA, Keshavarzian A (2002). PKC-zeta prevents oxidant-induced iNOS upregulation and protects the microtubules and gut barrier integrity. Am J Physiol Gastrointest Liver Physiol.

[B47] Yang C-M, Chien C-S, Hsiao L-D, Luo S-F, Wang C-C (2002). Interleukin-1beta-induced cyclooxygenase-2 expression is mediated through activation of p42/44 and p38 MAPKS, and NF-kappaB pathways in canine tracheal smooth muscle cells. Cell Signal.

[B48] Beere HM (2004). "The stress of dying": the role of heat shock proteins in the regulation of apoptosis. J Cell Sci.

[B49] Antonsson B, Conti F, Ciavatta A, Montessuit S, Lewis S, Martinou I, Bernasconi L, Bernard A, Mermod JJ, Mazzei G, Maundrell K, Gambale F, Sadoul R, Martinou JC (1997). Inhibition of Bax channel-forming activity by Bcl-2. Science.

[B50] Donovan M, Cotter TG (2004). Control of mitochondrial integrity by Bcl-2 family members and caspase-independent cell death. Biochim Biophys Acta.

[B51] Boatright KM, Salvesen GS (2003). Mechanisms of caspase activation. Curr Opin Cell Biol.

[B52] Beere HM, Green DR (2001). Stress management – heat shock protein-70 and the regulation of apoptosis. Trends Cell Biol.

[B53] Tepperman BL, Jacobson ED, Johnson LR (1994). Circulatory factors in gastric mucosal defense and repair. Physiology of the Gastroentestinal Tract.

